# *Clostridioides difficile* infection in pediatric inflammatory bowel disease: current understanding and clinical challenges

**DOI:** 10.3389/fped.2025.1753289

**Published:** 2026-01-20

**Authors:** Maria Rogalidou

**Affiliations:** Division of Gastroenterology & Hepatology, First Department of Paediatrics National and Kapodistrian University of Athens “Agia Sophia” Children’s Hospital, Athens, Greece

**Keywords:** children, clostridioides difficile infection, colonization, Crohn's disease, dysbiosis, inflammatory bowel disease, ulcerative colitis

## Abstract

*Clostridioides difficile* infection (CDI) represents a significant and increasingly recognized complication in children with inflammatory bowel disease (IBD), contributing to prolonged hospitalization and risk of adverse outcomes. Children with IBD are particularly susceptible due to frequent antibiotic exposure, healthcare system contact, immunosuppressive therapy, and underlying gut dysbiosis, all of which promote colonization and toxin-mediated intestinal injury. Distinguishing CDI from an IBD flare is challenging, as gastrointestinal symptoms and systemic inflammation overlap, and asymptomatic toxigenic colonization is common. Management recommendations for pediatric IBD-associated CDI are largely extrapolated from adult studies, with prompt initiation of targeted antibiotics being critical. Immunosuppressive therapy is generally continued, with escalation considered if diarrhea persists despite CDI-directed therapy. Fecal microbiota transplantation (FMT) has emerged as a safe and promising option for recurrent CDI in children with IBD, although careful patient selection, donor choice, and timing remain crucial. Key challenges persist in differentiating true CDI from IBD flares, understanding the clinical impact of asymptomatic colonization, and optimizing microbiome-targeted interventions. Future research should prioritize biomarker-driven diagnosis, individualized therapeutic strategies, and longitudinal evaluation of microbiome-based treatments to improve outcomes in pediatric patients with concurrent CDI and IBD.

## 1 Introduction

*Clostridioides difficile*, formerly known as *Clostridium difficile*, is a Gram-positive, spore-forming, anaerobic bacillus (1). It is well recognized for its ability to produce potent toxins and represents the leading cause of antibiotic-associated diarrhea worldwide. The clinical spectrum of *C. difficile* infection (CDI) ranges from asymptomatic colonization to mild diarrhea, and in severe cases, may progress to pseudomembranous colitis or toxic megacolon, potentially leading to septic shock and increased mortality. In one study, *C. difficile* was identified as the most frequently reported pathogen, accounting for 12.1% of healthcare-associated infections (2). In contrast to adult populations, CDI in children is more frequently community-acquired, representing approximately three-quarters of all pediatric cases (3). The infection is particularly prevalent among specific pediatric subgroups, including infants, oncology patients, and immunocompromised individuals. Moreover, CDI is notably more common and clinically significant in children with inflammatory bowel disease (IBD), often associated with exacerbation of intestinal inflammation, more severe disease manifestations, and challenges in managing the underlying condition.

## 2 Characteristics of microorganism

### 2.1 Distribution of *C. difficile*

*C. difficile* is widely distributed in nature and has been isolated from diverse environments, including water, soil, food products, and both domestic and farm animals, as well as household settings (4). Within healthcare facilities, *C. difficile* has been recovered from the hands of patients and healthcare personnel, hospital surfaces, medical equipment (5), and even hospital pet therapy dogs (6). The organism's ability to form robust spores contributes significantly to its persistence in both community and healthcare settings. These dormant spores exhibit remarkable resistance to environmental stressors such as heat, acid, antibiotics, and disinfectants, thereby facilitating environmental survival and transmission. Upon reaching the intestinal tract, the spores germinate into vegetative cells capable of producing toxins that drive the pathogenesis of *C. difficile* infection (CDI).

### 2.2 Toxin production by *C. difficile*

Clinical manifestations are related to intestinal damage by toxins. *C. difficile* produces two major toxins, TcdA and TcdB, encoded by the tcdA and tcdB genes within the pathogenicity locus (PaLoc). TcdA primarily functions as an enterotoxin, promoting intestinal inflammation and fluid secretion, whereas TcdB is a potent cytotoxin that induces epithelial cell death and mucosal damage (7, 8). Both toxins glucosylate Rho family GTPases (Rho, Rac, and Cdc42), disrupting the actin cytoskeleton, impairing tight junctions, and triggering apoptosis. Importantly, some strains express only TcdB yet still cause severe disease, highlighting TcdB as the principal virulence determinant. A subset of epidemic strains also produces a binary toxin (CDT), encoded by cdtA and cdtB, which ADP-ribosylates actin, leading to cytoskeletal disruption and the formation of microtubule-based protrusions that may enhance bacterial adherence. Although its precise role remains under investigation, CDT is associated with increased disease severity and recurrence. Toxin expression is tightly regulated within the PaLoc by tcdR (positive regulator), tcdC (negative regulator), and tcdE (involved in secretion), and is further influenced by environmental factors such as nutrient availability, antibiotic exposure, and pH (7–9).

### 2.3 Non-toxigenic *C. difficile* (NTCD) Strains

Non-toxigenic *C. difficile* (NTCD) strains lack the genes encoding toxins A (tcdA) and B (tcdB) and are therefore generally considered non-pathogenic. Despite their inability to produce toxins, NTCD can colonize the gastrointestinal tract, particularly in children, without causing clinical disease. These strains retain the ability to form spores, allowing them to persist in both the environment and the host gut. NTCD can establish asymptomatic colonization, which may be transient or, in some cases, long-term. Although they lack toxin genes, NTCD strains are genetically diverse and may carry other virulence-associated genes. Asymptomatic colonization with NTCD is frequently observed in healthy individuals, especially in pediatric populations, without associated clinical symptoms. Understanding these mechanisms is particularly relevant in pediatric populations, where *C. difficile* infection can range from asymptomatic colonization to life-threatening colitis, and where recurrence remains a significant clinical challenge (10).

## 3 Pathogenesis

The pathogenesis of CDI is multifactorial, involving both bacterial virulence and host factors. While toxin production is necessary for disease, it is not sufficient on its own; host susceptibility is critical. Toxins induce intestinal epithelial injury and inflammation, which drive clinical symptoms. Host immune responses modulate disease severity, as individuals with low or undetectable antitoxin antibodies are more likely to develop symptomatic infection, whereas post-infection antitoxin responses confer protection against recurrence (7–9). Colonization with non-toxigenic *C. difficile* strains may protect against disease by competitive inhibition of toxigenic strains (10). Additional determinants of susceptibility include the variable expression of intestinal toxin receptors and the inhibitory effects of intestinal bile acids on spore germination and bacterial growth (11).

The pathogenesis of pediatric IBD—encompassing Crohn's disease (CD), ulcerative colitis (UC), and IBD-unclassified (IBD-U)—involves a complex interplay among genetic susceptibility, environmental influences, alterations in the intestinal microbiome, and dysregulated mucosal immune responses. These interactions lead to a loss of tolerance to intestinal commensal bacteria, resulting in chronic intestinal inflammation (12).

Children with IBD have been shown to exhibit altered fecal bile acid profiles (13, 14), characterized by higher levels of primary bile acids and reduced levels of secondary bile acids (15, 16). Bile acids play a critical role in the pathogenesis of CDI, as primary bile acids promote spore germination into the vegetative, toxin-producing form of the bacterium, while secondary bile acids inhibit *C. difficile* growth (17).

Some other compounds have been associated with both intestinal inflammation and metabolic pathways utilized by *C. difficile* (13). In a multi-omic analysis of stool samples from children with IBD, distinct metabolic profiles were found to differentiate patients with and without CDI (13). Several metabolites, including taurine and isocaproyltaurine, were elevated in CDI-positive samples compared to non-CDI samples.

### 3.1 Does *C. difficile* plays role in inflammatory bowel disease development?

While CDI is often associated with disease relapses, a substantial number of pediatric cases are identified at the time of IBD diagnosis. CDI and IBD are interrelated conditions, where CDI can worsen IBD symptoms, and having IBD increases the risk of developing CDI.

*C. difficile* may contribute to the development of IBD in susceptible individuals, although its role is not fully established. Several studies have reported a higher prevalence of toxigenic *C. difficile* in newly diagnosed pediatric and adult patients with IBD, even in the absence of prior antibiotic exposure, suggesting that colonization might precede or promote disease onset (18–20). *Clostridium* species contribute to the pathogenesis and progression of IBD through both protective and pathogenic mechanisms. Commensal species, such as *Clostridium butyricum*, produce short-chain fatty acids (SCFAs) like butyrate that strengthen the intestinal barrier, enhance mucus production, and modulate immune responses by promoting regulatory T cell differentiation, thereby maintaining gut homeostasis and protecting against inflammation, or inversely their disbalance can promote the gut inflammation (21–23). The major toxins produced by *C. difficile*, TcdA and TcdB, disrupt the intestinal epithelial barrier, increase gut permeability, and trigger pro-inflammatory cytokine release, which may favor chronic intestinal inflammation (24, 25). Overall, while *C. difficile* appears to play a role in promoting intestinal inflammation and may act as a risk factor for IBD in predisposed individuals, it is not considered a primary cause of the disease. In contrast, pathogenic species, particularly *C. difficile*, disrupt epithelial integrity through toxin production, trigger pro-inflammatory Th1/Th17 immune responses, and can precipitate disease flares in susceptible individuals (26–29). Dysbiosis favoring pathogenic clostridia amplifies mucosal inflammation and exacerbates disease severity, highlighting the dual and critical influence of these bacteria in both the onset and progression of IBD (30–32).

**Figure 1:**
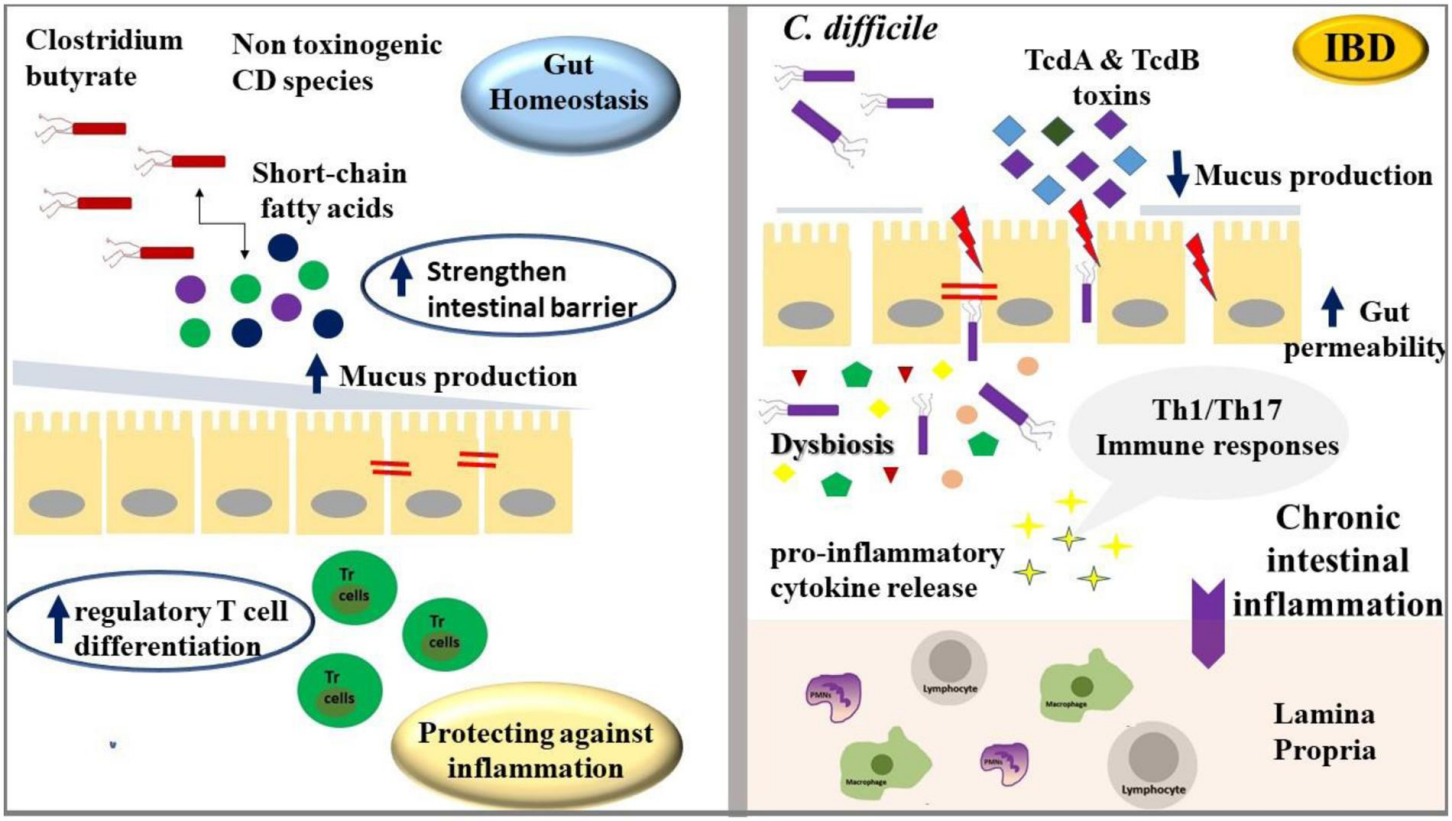
Clostridium species exert both protective and pathogenic roles in IBD. Commensal strains like *C. butyricum* produce butyrate that supports epithelial integrity and immune regulation, while pathogenic *C. difficile* releases toxins that disrupt the barrier and drive inflammation. Dysbiosis favoring pathogenic clostridia exacerbates mucosal inflammation and disease severity.

The potential role of *Clostridioides/Clostridium* species in gut homeostasis and in inflammatory bowel disease development appears in Figure 1.

## 4 Epidemiology

Although CDI is generally more prevalent in adults than in children, those with IBD demonstrate similarly high rates of CDI. The pathogenesis and predisposing factors for CDI in pediatric IBD may differ from those seen in adults. Possible explanations include the higher rates of asymptomatic C. difficile colonization observed in children, differences in IBD presentation and distribution, and the ongoing development and variability of the intestinal microbiota during childhood (33).

CDI is an increasingly significant pediatric health concern, with approximately 20,000 cases reported annually and a growing community-associated infection trend (3).

According to active population-based surveillance conducted in 2022, the rate of CDI among children younger than 18 years in the United States was estimated to be 29.1 cases per 100,000 population. About three-quarters of these infections occurred in the community rather than in hospitals (34).

Children with IBD experience a higher incidence of symptomatic CDI and an increased risk of recurrence compared with the general pediatric population (35–39). Hospitalizations related to CDI are rising among pediatric IBD patients (38). In children hospitalized with IBD, CDI is associated with longer hospital stays, greater need for parenteral nutrition, and more frequent blood transfusions (38). Additionally, CDI has been linked to exacerbations of IBD in this population (35, 37, 38).

Research indicates that approximately 3%–7% of children with CDI progress to fulminant disease, which may present as hypotension or shock, ileus, or toxic megacolon (40, 41). Among hospitalized children with CDI, there has been no observed change over time in the rates of colectomy or mortality (42–44). However, CDI in pediatric IBD is linked to a higher likelihood of intestinal surgery (35, 45). In a case-control cohort study, 8% of children with IBD who developed CDI underwent abdominal surgery within six months, compared with 3% of children with IBD without CDI (*p* < 0.01) (46). Although CDI necessitating colectomy is uncommon in children (0.3% of all pediatric CDI cases), the majority (74.8%) of colectomies reported in a large pediatric CDI database were performed in children with IBD (42). This study reported a decline in the incidence of CDI among hospitalized children, representing a shift from previous observations. However, this decrease may have been influenced by changes in diagnostic testing practices. The study also found that CDI occurred most frequently in children with chronic gastrointestinal conditions (36%) and malignancies (32%), respectively (42).

Data from a large hospital discharge database, which included over 20,000 pediatric cases of CDI, showed that children diagnosed with IBD had an approximately eleven-fold higher likelihood of developing CDI compared with those without IBD (43). Likewise, another investigation analyzing hospital discharge records from a single U.S. state reported an incidence rate ratio of 12.7 for CDI in children with IBD when compared with healthy peers. The reported prevalence of CDI in pediatric IBD patients varies widely, ranging from 3.5% to 69% (12).

In comparison, adult patients with IBD demonstrated a lower relative risk, with an incidence rate ratio of 4, indicating that pediatric IBD patients are disproportionately more susceptible to CDI than adults with the same condition (47).

At present, IBD is recognized as one of the leading comorbidities that heighten the risk of CDI in children (36, 47, 48). Multiple studies have examined the incidence of CDI in pediatric patients with IBD in comparison to healthy children.

In adults with IBD, colonic involvement—particularly ulcerative colitis—is consistently associated with an increased risk of CDI (14, 49). In contrast, pediatric studies show more variable findings, with CDI risk in children appearing to relate more to disease severity and intestinal dysbiosis than to colonic disease extent alone (18, 37, 38, 50–52).

In children with IBD, the occurrence of CDI is associated with worse outcomes, including exacerbation of disease and longer hospital stays. Data from two large hospital discharge studies showed that CDI led to an increase in the average hospitalization duration from 6 days to 8 days in this population (38, 53).

These children often need intensification of their IBD treatment. In a retrospective study of 111 pediatric patients with IBD and CDI, 67% required escalation of therapy, defined as the addition of an immunomodulator or biologic agent to their existing regimen (46). Likewise, a population-based study from a single Canadian province found that children with IBD and CDI were more likely to require systemic steroids or tumor necrosis factor (TNF) inhibitors in the future (48). It remains uncertain whether CDI actively worsens the course of IBD or if it primarily occurs in children who already have a more severe IBD phenotype, placing them at greater risk for complications. Regardless, the identification of CDI in a pediatric patient with IBD should prompt careful monitoring, as it may signal a more complex disease trajectory. Overall, children with IBD are more susceptible to CDI and experience poorer outcomes, highlighting the need for enhanced strategies for prevention, early diagnosis, and effective management (14). rCDI is defined as a new episode of CDI occurring within eight weeks of the initial infection. Reported recurrence rates among pediatric IBD populations range from 25% to 34% (39, 46, 48).

## 5 Risk factors

### 5.1 Antibiotic exposure

Exposure to antibiotics is the primary risk factor for both healthcare-associated and community-associated CDI in children (54). Many recognized risk factors for CDI in the general population—such as hospitalization and antibiotic use—are also more common in patients with IBD, which may contribute to their increased susceptibility. Antibiotics can disrupt the normal gastrointestinal microbiome, creating conditions that favor the overgrowth of *C. difficile* and enhanced toxin production (55). Although any antibiotic can elevate the risk of CDI, the degree of risk varies by antibiotic class (56). In children, the risk of CDI varies by antibiotic class. Clindamycin and third-generation cephalosporins carry the highest risk, while broad-spectrum penicillins, fluoroquinolones, and carbapenems pose a moderate risk. Tetracyclines and aminoglycosides are rarely associated with CDI in pediatric patients. These differences reflect both the spectrum of activity and the frequency of use in children, with high-risk agents often disrupting gut microbiota more profoundly (55, 56).

### 5.2 The use of proton pump inhibitors (PPIs)

In children, the use of PPIs, and to a lesser extent histamine-2 receptor antagonists (H2RAs), has been linked to an elevated risk of CDI (57). However, studies examining CDI in children with IBD has found no significant link between the use of acid-suppressing medications and the risk of developing CDI (37, 46, 50).

### 5.3 Microbiome

A diverse intestinal microbiota provides protection against the establishment of potential pathogens, including *C. difficile*—a mechanism known as colonization resistance (58). Individuals with IBD exhibit a dysbiotic gut microbiome, characterized by reduced microbial diversity and altered microbial composition (59, 60). This disruption creates an intestinal environment that favors the colonization and spore germination of *C. difficile*. Comparative studies examining the gut microbiota of patients with IBD, with and without CDI have revealed distinct microbial differences between these groups.

Gut dysbiosis—marked by reduced microbial diversity and altered composition—creates conditions that favor C. difficile colonization and spore germination. Comparative studies show that IBD patients with CDI often exhibit depletion of butyrate-producing Firmicutes, which are important for epithelial integrity, along with expansion of opportunistic Proteobacteria. These microbial alterations not only weaken colonization resistance but may also intensify mucosal inflammation, increasing susceptibility to CDI (59, 60).

### 5.4 Medications

Although, medications such as antibiotics and PPIs are recognized risk factors for CDI in the general population, can also increase CDI risk in patients with IBD, however, many patients with IBD develop CDI even in the absence of these exposures (61).

CDI can exacerbate IBD symptoms, and patients with more extensive disease may have a higher risk of CDI, often necessitating greater use of immunosuppressive agents and corticosteroids. Importantly, this does not imply that these therapies themselves directly increase CDI risk.

There is conflicting evidence regarding whether anti-inflammatory and immunomodulatory therapies used for IBD contribute to an increased risk of CDI. In both the general population and adults with IBD, the use of immunosuppressive agents and corticosteroids has been associated with a higher risk of CDI (62). However, a recent meta-analysis of 14 studies examining CDI in children with IBD found no significant association between CDI risk and the use of steroids, immunomodulators, or biologic agents, suggesting that pediatric CDI may have distinct clinical characteristics (50). Interestingly, only 5-aminosalicylic acid (5-ASA) therapy was linked to an increased risk of CDI in children with IBD (odds ratio = 1.95, 95% CI 1.26–3.0) (50), though the underlying mechanism for this association remains unclear.

### 5.5 IBD type and disease severity

In adult populations, both *C. difficile*–associated disease (CDAD) and colonization with toxigenic *C. difficile* are more frequently observed in UC compared with CD (33, 63). In pediatric populations, however, studies generally report no significant difference in CDI or toxigenic *C. difficile* colonization between UC and CD (18, 35, 37, 47). Nevertheless, colonic involvement in pediatric IBD is consistently associated with an increased risk of CDI (18, 35, 47).

Beyond IBD subtype, disease severity is a major factor influencing CDI risk. CDI is more frequently observed and often more severe in patients with active or severe IBD (18, 35, 47). Pediatric studies suggest that worsening microbial dysbiosis associated with severe IBD may reduce colonization resistance, facilitating overgrowth of *C. difficile* (33). As a result, some investigators have proposed that detection of toxigenic *C. difficile* in IBD may serve primarily as a marker of underlying disease activity rather than indicating a distinct infectious process.

### 5.6 Other

The imbalance in bile acid metabolism (11) and other metabolomes (13) may contribute to the increased susceptibility of patients with IBD to CDI. Consistent with this, children with IBD and active CDI have been observed to have elevated fecal primary bile acids and diminished secondary bile acids (13, 64). These metabolic alterations may also hold potential for the development of novel diagnostic approaches for CDI.

## 6 Clinical presentation

The clinical presentation of CDI in children with IBD is often difficult to distinguish from an IBD flare, as both conditions share overlapping gastrointestinal and systemic manifestations. CDI can exacerbate underlying intestinal inflammation, leading to disease worsening, hospitalization, and recurrence (65). The severity of the clinical picture of CDI in children appears in Table 1 (65).

**Table 1:** The severity of clinical picture of *C. difficile* infection in children.

Mild	Moderate	Severe*	Fulminant
Afebrile	Fever	Fever	Hypotension
Diarrhea (without systemic findings)	Profuse diarrhea	Profuse diarrhea	Shock
	Abdominal pain	Abdominal pain and tenderness	Ileus
			Toxic megacolon
		Abdominal distention	
		Leukocytosis (white blood cells ≥15,000 cells/microL)	
		Elevated age-adjusted creatinine level	
		Pseudomembranous colitis	

**Indications for hospitalization:** Children with severe or fulminant disease should be hospitalized.

* There is no consensus definition of severe *C. difficile* infection in children. Determination of disease severity should be guided by clinician judgment (66).

### 6.1 Gastrointestinal manifestations

Diarrhea is the most common symptom, ranging from mild to profuse watery stools. Abdominal pain, cramping, and bloody diarrhea are frequently reported, particularly in patients with ulcerative colitis. Additional symptoms such as urgency, tenesmus, and rectal discomfort reflect lower gastrointestinal tract involvement.

### 6.2 Systemic symptoms

Systemic manifestations, including fever, malaise, and dehydration, may occur in moderate to severe cases. Laboratory abnormalities such as leukocytosis, anemia, elevated C-reactive protein (CRP), and hypoalbuminemia are common but non-specific and may also reflect active IBD.

### 6.3 Recurrent infection

Recurrent CDI (rCDI) represents a significant clinical challenge in children with IBD, largely due to persistent intestinal dysbiosis, prior antibiotic exposure, and ongoing immunosuppressive therapy. rCDI is defined as a new episode of CDI occurring within eight weeks of the initial infection. Reported recurrence rates among pediatric IBD populations range from 25% to 34% (39, 46, 48).

### 6.4 Diagnostic considerations

Given the considerable clinical overlap between CDI and IBD relapse, stool testing for *C. difficile* toxins or genes should be performed in all pediatric patients with IBD presenting with new or worsening diarrhea, particularly following recent antibiotic exposure or hospitalization. Prompt identification and targeted treatment are essential to prevent severe outcomes and reduce the risk of recurrence (67).

### 6.5 Asymptomatic colonization

Distinguishing a true CDI from an IBD flare in children can be challenging due to the relatively high rate of *C. difficile* colonization in this population. Colonization refers to the presence of toxigenic *C. difficile* in the gut without causing symptoms (66). Studies have reported that between 10% and 25% of children with IBD may carry *C. difficile* asymptomatically, complicating the interpretation of positive test results and the identification of active infection (36, 49, 68).

## 7 Diagnostic testing for CDI

Several diagnostic methods are available for detecting CDI, including enzyme immunoassays (EIA) for toxins A and B, EIA for glutamate dehydrogenase (GDH), nucleic acid amplification tests (NAATs), toxigenic culture, and next-generation sequencing (NGS). However, no single test is entirely accurate, as each varies in sensitivity and specificity (61, 69–73).

The NAAT, a PCR-based assay, detects toxin-producing genes of *C. difficile* and offers high sensitivity, but it cannot distinguish colonization from active infection. Similarly, the GDH EIA, which identifies the GDH enzyme produced by both toxigenic and non-toxigenic strains, serves as a highly sensitive screening test. The toxin EIA, which detects toxins A and/or B, is often used as a confirmatory test due to its lower sensitivity and a false-negative rate of up to 30% (61, 69–71, 73).

Advanced methods such as toxigenic culture and next-generation sequencing (NGS) can accurately identify *C. difficile* and its toxin genes, but their high cost and long turnaround time limit their use in routine clinical practice (72). The diagnostics tests for CDI appear in Table 2.

**Table 2:** The diagnostics tests for *C. difficile* infection.

Diagnostic test-method	Target/Principle	Sensitivity & Specificity	Advantages	Limitations	Clinical Use
Nucleic Acid Amplification Test (NAAT/PCR)	Detects toxin-producing genes (*tcdA*, *tcdB*)	High sensitivity and specificity	Rapid and accurate detection of toxigenic *C. difficile*	Cannot distinguish colonization from true infection	Commonly used as an initial or confirmatory test
Glutamate Dehydrogenase (GDH) Enzyme Immunoassay (EIA)	Detects GDH enzyme in both toxigenic and non-toxigenic strains	High sensitivity, low specificity	Fast, inexpensive, good screening tool	Positive results require confirmatory toxin testing	Used as a screening step in a multistep algorithm
Toxin A/B Enzyme Immunoassay (EIA)	Detects toxins A and/or B	Moderate sensitivity (∼70%), high specificity	Identifies toxin presence, confirming active infection	Up to 30% false-negative rate	Used as a confirmatory step after NAAT or GDH
Toxigenic Culture	Culture of *C. difficile* followed by toxin testing	High sensitivity and specificity (gold standard)	Provides isolates for epidemiologic studies	Time-consuming and labor-intensive	Research or complex diagnostic cases
Next-Generation Sequencing (NGS)	Detects genetic sequences of *C. difficile* and toxin genes	Very high accuracy	Offers detailed genetic information	Expensive and slow turnaround	Used in research and outbreak investigations

The diagnosis of CDI remains a clinical and laboratory composite, where stool testing must be interpreted in the context of symptoms (e.g., diarrhea), pretest probability, and other causes of gastrointestinal illness. Laboratory assays alone — whether PCR, toxin EIA, or advanced sequencing — cannot by themselves definitively distinguish colonization from active infection in most clinical settings.

**Figure 2:**
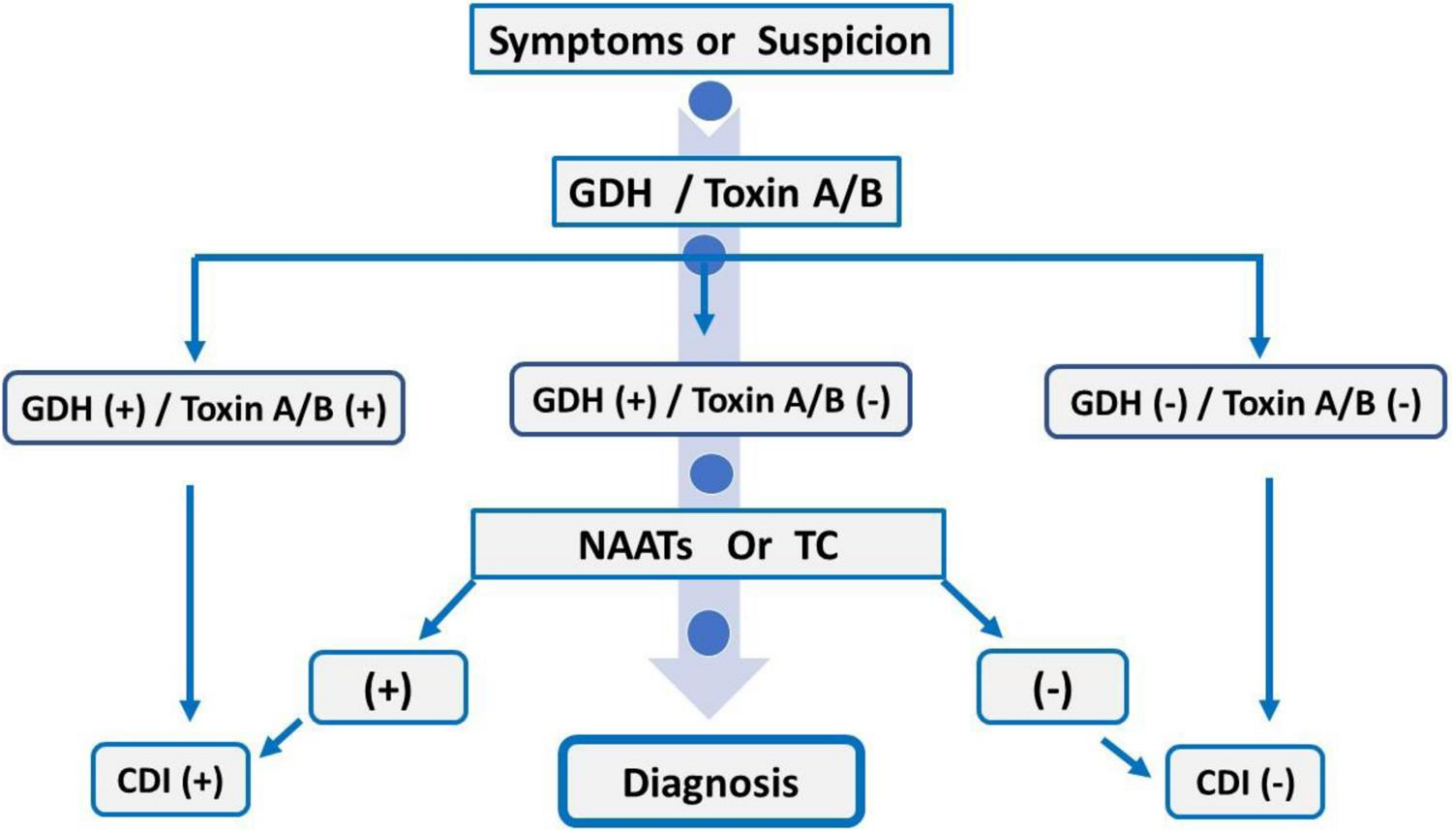
The two-step approach for diagnosing CDI. EIA for GDH and toxin A/B. CDI, clostridium difficile infection; EIA, enzyme immunoassay; GDH, glutamate dehydrogenase; NAAT, nucleic acid amplification test; TC, toxigenic culture.

Current guidelines for diagnosing CDI recommend either: (A) a two-step testing strategy, combining different diagnostic assays, or (B) the use of a highly sensitive test such as NAAT, applied only in patients who meet strict clinical criteria. The purpose of these approaches is to improve diagnostic accuracy by enhancing both sensitivity and specificity while reducing false-positive results caused by asymptomatic *C. difficile* colonization. A diagnostic algorithm appears in Figure 2.

## 8 Management

### 8.1 Treatment of CDI in pediatric patients with IBD

Although guidelines exist for the management of CDI in children (71, 77, 78), there are currently no recommendations specifically addressing CDI in pediatric patients with IBD therefore, existing recommendations are primarily extrapolated from adult studies and treatment data (14, 63). Following diagnosis, prompt initiation of antibiotic therapy is critical to prevent progression and complications of CDI. Evidence from a retrospective observational study in adults patients with IBD demonstrated that those with UC and CDI who were treated with oral vancomycin experienced fewer readmissions and shorter hospital stays compared with those receiving metronidazole (33, 63). Based on these findings, it has been suggested that oral vancomycin may be considered first-line therapy for CDI in adults with IBD, although direct pediatric data are lacking. Fidaxomicin may be considered as an alternative option for the management. In cases of severe or fulminant disease, rectal vancomycin can be administered when oral delivery is not feasible, and intravenous metronidazole may be used as adjunctive therapy. For recurrent CDI, current guidelines support treatment with pulse-tapered oral vancomycin or oral vancomycin followed by rifaximin or fidaxomicin. Bezlotoxumab, a monoclonal antibody against C. difficile toxin B, can be given as a single IV dose (10 mg/kg) alongside antibiotics to reduce recurrence in adults at high risk; it does not treat active infection. It is generally safe, though caution is advised in patients with heart failure. Guidelines recommend considering bezlotoxumab for patients with prior CDI, older age, immunocompromise, or severe infection (74). The pediatric doses for CDI infection appear in Table 3.

**Table 3:** Pediatric CDI treatment dosing.

Drug	Age/Weight Group	Route	Dose	Frequency	Duration
Metronidazole	All pediatric ages	PO	7.5 mg/kg per dose	TID or QID	10 d
			(max 500 mg)		
Metronidazole	All pediatric ages	IV	10 mg/kg	TID	10 d
			(max 500 mg)		
Vancomycin	All pediatric ages	PO	10 mg/kg per dose	10 d	10 d
			(max 125 mg)		
Vancomycin tapered and pulsed	All pediatric ages	PO	10 mg/kg per dose	QID 10–14 d,	
				then BID × 7 d,	
			(max 125 mg)	then once daily × 7 d,	
				then every 2–3 d for 2–8 wk	
Vancomycin*; followed by rifaximin chaser	All pediatric ages*	PO	10 mg/kg per dose (max 125 mg)	QID; TID	10 d; 20 d
			No pediatric dosing for rifaximin (max 400 mg)		
Vancomycin	1–3 years—50 mL	IR (administered by retention enema)	10 mg/kg per dose in normal saline (maximum dose 500 mg in 100 mL normal saline)	QID	10 d
	4–10 years—75 mL				
	>10 years—100 mL				
Fidaxomicin	≥6 months and ≥4 kg	PO	4 to <7 kg:80 mg	BID	10 d
			7 to <9: 120 mg		
			9 to <12.5 kg 160 mg ≥12.5 kg 200 mg		

* Fidaxomicin not recommended for children <12.5 kg.

Importantly, immunosuppressive therapy should not be discontinued during CDI management. Because CDI and IBD flares share overlapping clinical features, careful and ongoing assessment for active colitis is essential. If diarrhea persists without improvement by day 3–4 of CDI-targeted antibiotic therapy, escalation of immunosuppressive treatment should be considered to address potential IBD activity (63). Pediatric CDI treatment Guidelines and Management: First-line, Alternatives, and Recurrence appear in Table 4.

**Table 4:** Pediatric CDI treatment guidelines and management: first-line, alternatives, and recurrence.

Clinical Scenario	ASID 2025 (Australia/NZ) (74)	IDSA/SHEA 2017 (USA) (73)	ECDC 2021 (Europe) (75)	Pediatric IBD Considerations
Initial Episode—Non-severe	**First-line:** Vancomycin (oral)	**First-line:** Metronidazole (mild) or Vancomycin (oral)	**First-line:** Metronidazole or Vancomycin (oral)	Vancomycin preferred in IBD due to higher risk of complications; fidaxomicin reduces recurrence
	**Alternative:** Fidaxomicin (≥12.5 kg, high-risk)			
		**Alternative:** Fidaxomicin (recurrent/high-risk)	**Alternative:** Fidaxomicin (recurrent/high-risk)	
Initial Episode—Severe/Complicated	**First-line:** Vancomycin (oral or rectal) + Metronidazole (IV)	**First-line:** Vancomycin (oral or rectal) + Metronidazole (IV)	**First-line:** Vancomycin (oral or rectal) + Metronidazole (IV)	Close monitoring required; immunosuppression or IBD may worsen severity
	**Alternative:** Fidaxomicin for high-risk	**Alternative:** Fidaxomicin for high-risk/recurrent	**Alternative:** Fidaxomicin for high-risk/recurrent	
First Recurrence	**First-line:** Repeat initial therapy based on severity	**First-line:** Repeat initial therapy based on severity	**First-line:** Repeat initial therapy based on severity	Adjust therapy based on severity and IBD activity
	**Alternative:** Fidaxomicin (if not used previously)	**Alternative:** Fidaxomicin (if not used previously)	**Alternative:** Fidaxomicin (if not used previously)	
Second or Subsequent Recurrences	**First-line:** Vancomycin tapered/pulsed or FMT	**First-line:** Vancomycin tapered/pulsed or FMT	**First-line:** Vancomycin tapered/pulsed or FMT	FMT in pediatric IBD should be performed in specialized centers (76)**;** fidaxomicin reduces recurrence risk; bezlotoxumab rarely used/off-label
	**Alternative:** Fidaxomicin (if not used previously)	**Alternative:** Fidaxomicin (if not used previously)	**Alternative:** Fidaxomicin (if not used previously)	
	**Special/Off-label:** Bezlotoxumab in select high-risk cases	**Special/Off-label:** Bezlotoxumab for high-risk	**Special/Off-label:** Bezlotoxumab for high-risk	
Prevention/Infection Control	Contact precautions, hand hygiene with soap and water, environmental cleaning with sporicidal agents	Contact precautions, hand hygiene with soap and water, environmental cleaning with sporicidal agents	Contact precautions, hand hygiene with soap and water, environmental cleaning with sporicidal agents	Limit unnecessary antibiotics during IBD flares
Special Populations	Immunocompromised children: same principles; monitor closely	Immunocompromised children: same principles; monitor closely	Immunocompromised children: same principles; monitor closely	IBD patients often immunosuppressed; coordination with gastroenterology essential

### 8.2 Response to treatment

The clinical response to therapy for CDI is primarily assessed based on symptomatic improvement. In cases of mild to moderate disease, most patients demonstrate clinical improvement within 48–72 h following the initiation of appropriate antibiotic treatment. However, complete resolution of diarrhea may take four to five days, especially in patients treated with metronidazole or carriers of NAP1/BI/027 strain even with effective therapy (65). Following completion of therapy, routine testing for *C. difficile* eradication (“test of cure”) is not recommended, as clinical improvement should guide treatment success (65, 71).

### 8.3 Surgical therapy for fulminant disease

Surgical intervention, such as subtotal colectomy or diversion ileostomy, may be necessary as a life-saving measure in children with fulminant CDI who develop toxic megacolon, colonic perforation, acute abdomen, or septic shock. However, the indications for surgery in pediatric patients with severe or fulminant CDI are not well defined, and current practice is largely guided by adult experience and case-based clinical judgment (65).

### 8.4 Efficacy and outcomes of FMT in pediatric patients with IBD

Fecal microbiota transplantation (FMT) is increasingly utilized in pediatric gastroenterology, primarily for the treatment of recurrent *C. difficile* infection (rCDI). Beyond rCDI, growing evidence underscores the pivotal role of the gut microbiome in health and disease, prompting investigation of fecal microbiota transplantation (FMT) as a therapeutic approach for other conditions, including inflammatory bowel disease (IBD), graft-vs.-host disease, neuropsychiatric disorders, and metabolic syndrome. Recent ESPGHAN and NASPGHAN guidelines provide recommendations on FMT use in children with CDI especially with IBD, offering guidance on patient selection, procedural protocols, and safety considerations (75).

FMT is generally well tolerated, but children with IBD or compromised immune systems may experience more pronounced side effects. There is a potential for triggering IBD flares, and the long-term effects on the immune system remain unclear, highlighting the need for careful monitoring. These factors emphasize the importance of studying FMT specifically in pediatric populations. Recent systematic reviews call for well-designed studies to evaluate its effectiveness, monitor adverse events, and establish safe practices for use in children (76).

A multicenter retrospective cohort study demonstrated comparable efficacy of FMT for rCDI in children with and without IBD (76% vs. 81%, *p* = 0.17). Within the IBD cohort, treatment success was more likely when fresh donor stool was used, in patients without active diarrhea at the time of the procedure, and when the interval between CDI diagnosis and FMT was shorter. Post-procedure, 13% of children with IBD required hospitalization within three months; however, it remains unclear whether these events were attributable to FMT, the underlying disease, or both. These findings suggest that FMT is a viable therapeutic option for rCDI in pediatric IBD, though careful patient selection and monitoring are warranted (79).

## 9 Gaps and future directions

A major clinical challenge in pediatric IBD is distinguishing true CDAD from an IBD flare with *C. difficile* colonization, particularly since toxigenic colonization is more common in children. It remains uncertain whether *C. difficile* acts as a trigger of inflammation or merely reflects underlying disease activity. Future studies evaluating inflammatory and immune biomarkers may help differentiate these entities.

Given the high treatment failure rates and adverse outcomes associated with CDI in pediatric IBD, research is needed to determine whether more aggressive or tailored treatment approaches are warranted. The impact of asymptomatic *C. difficile* colonization on IBD progression is also unclear and should be investigated through large prospective studies. Finally, microbiome-based therapies, such as FMT, hold promise for improving outcomes in children with concurrent CDI and IBD.

## 10 Conclusions

Children with IBD are particularly vulnerable to both initial and recurrent *C. difficile* infections, likely due to underlying gut inflammation, altered microbiota, and frequent healthcare exposure. The overlap of CDI symptoms with IBD flares, along with the limitations of current diagnostic tests, creates a significant challenge in accurately identifying infection. CDI in pediatric patients with IBD is associated with longer hospitalizations, increased need for surgical intervention, and potential escalation of IBD therapy, though it remains unclear whether CDI worsens disease outcomes or reflects more severe underlying IBD. Antibiotics remain the primary treatment, but high recurrence rates highlight the potential role of alternative therapies, such as fecal microbiota transplantation. Given the reliance on adult data and the limitations of retrospective studies, prospective pediatric research is essential to clarify risk factors, optimize diagnosis, and guide effective treatment strategies for this population.
